# Determination and Occurrence of Phenoxyacetic Acid Herbicides and Their Transformation Products in Groundwater Using Ultra High Performance Liquid Chromatography Coupled to Tandem Mass Spectrometry

**DOI:** 10.3390/molecules191220627

**Published:** 2014-12-10

**Authors:** Sarah-Louise McManus, Mary Moloney, Karl G. Richards, Catherine E. Coxon, Martin Danaher

**Affiliations:** 1 Teagasc Environmental Research Centre, Johnstown Castle, Wexford, Ireland; E-Mail: sarahlouisemcmanus@gmail.com; 2 Department of Geology, Trinity College Dublin, Dublin 2, Ireland; E-Mail: cecoxon@tcd.ie; 3 Food Safety Department, Teagasc Food Research Centre, Ashtown, Dublin 15, Ireland; E-Mails: Mary.Moloney@teagasc.ie (M.M.); Martin.Danaher@teagasc.ie (M.D.)

**Keywords:** phenoxyacetic acid herbicides, chlorophenols, benzonitriles, water, UHPLC-MS/MS

## Abstract

A sensitive method was developed and validated for ten phenoxyacetic acid herbicides, six of their main transformation products (TPs) and two benzonitrile TPs in groundwater. The parent compounds mecoprop, mecoprop-p, 2,4-D, dicamba, MCPA, triclopyr, fluroxypr, bromoxynil, bentazone, and 2,3,6-trichlorobenzoic acid (TBA) are included and a selection of their main TPs: phenoxyacetic acid (PAC), 2,4,5-trichloro-phenol (TCP), 4-chloro-2-methylphenol (4C2MP), 2,4-dichlorophenol (DCP), 3,5,6-trichloro-2-pyridinol (T2P), and 3,5-dibromo-4-hydroxybenzoic acid (BrAC), as well as the dichlobenil TPs 2,6-dichlorobenzamide (BAM) and 3,5-dichlorobenzoic acid (DBA) which have never before been determined in Irish groundwater. Water samples were analysed using an efficient ultra-high performance liquid chromatography (UHPLC) method in an 11.9 min separation time prior to detection by tandem mass spectrometry (MS/MS). The limit of detection (LOD) of the method ranged between 0.00008 and 0.0047 µg·L^−1^ for the 18 analytes. All compounds could be detected below the permitted limits of 0.1 µg·L^−1^ allowed in the European Union (EU) drinking water legislation [1]. The method was validated according to EU protocols laid out in SANCO/10232/2006 with recoveries ranging between 71% and 118% at the spiked concentration level of 0.06 µg·L^−1^. The method was successfully applied to 42 groundwater samples collected across several locations in Ireland in March 2012 to reveal that the TPs PAC and 4C2MP were detected just as often as their parent active ingredients (a.i.) in groundwater.

## 1. Introduction

In order to fulfil the world’s growing demand for food, herbicide application to crops is necessary. Herbicides are a specific group of plant protection products (PPP) used to treat broad leaved weeds and other associated weeds which may reduce crop productivity. The phenoxyacetic acid herbicides are one of the most commonly used groups of PPPs because of their low cost, effectiveness and good water solubility [2]. They are widely used in agriculture and recreational areas such as golf courses and watercourses. In addition, phenoxyacetic acid herbicides readily degrade through biological and photolytic mechanisms, depending on the environmental conditions they are exposed to [3]. Chlorophenols are one of their main transformation products (TPs) and these can be more toxic than the parent product [4,5,6]. For example, 4-chloro-2-methylphenol (4C2MP), a transformation product (TP) of MCPA, mecoprop and mecoprop-p, persists in the environment, bioaccumulates and is toxic to aquatic organisms [7]. Table 1 shows the herbicides used in Ireland and determined by the method described in this paper, alongside their most notable TPs. Clausen *et al.* [8] and Holtze *et al.* [9] indicate that the TP 2,6-dichlorobenzamide (BAM) can be formed from dichlobenil while Jensen *et al.* [10] state that 3,5-dichlorobenzoic acid (DBA) can be formed by degradation of BAM and dichlobenil. Dichlobenil was widely used on watercourses for aquatic weed control and on non-agricultural areas [11], until it was banned following inclusion in part 2 of Annex I to regulation (EC) 689/2008 [12].

It is important to monitor for herbicides and their environmental TPs on an ongoing basis to evaluate water quality. The EU Water Framework Directive (WFD) states that by 2015 all water bodies must achieve “good status” [13]. Should groundwater bodies contain more than the drinking water limits from EU Council Directive 98/83/EC [1] (*i.e*., 0.1 µg·L^−1^ of a single pesticide or greater than or equal to 0.5 µg·L^−1^ of total pesticides within a single sample) then the water body of interest will not achieve “good status”. These permitted levels are also stipulated in the EU Groundwater Directive [14].

Several methods have been developed to detect in water the parent phenoxyacetic acid herbicides including 2,4-D, bentazone, bromoxynil, dicamba, triclopyr, and mecoprop [3,15,16,17,18,19,20,21]. In addition, methods have been developed to determine phenoxyacetic acid herbicide TPs such as TCP, 4C2MP and 2,4-dichlorophenol (DCP) in water [15,16,17,22]. However, no methods have yet been reported that analyse a wide range of phenoxyacetic acid herbicides and their associated TPs together. In addition, the benzonitrile TPs BAM and DBA, have been analysed separately to phenoxyacetic acid herbicides in stand-alone methods [10,16,20].

**Table 1:** The structures, chemical abstracts service (CAS) registry number of the active ingredients (a.i.) and transformation products (TPs) analysed by this analytical method.

Parent Active Ingredient (a.i.)	CAS Number	Transformation Product(s) (TP)	CAS Number
MCPA (4-chloro-2-methylphenoxy acetic acid)	94-74-6	Phenoxyacetic acid (PAC)	122-59-8
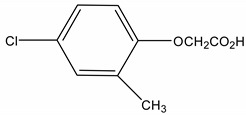		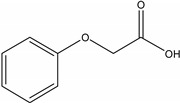	
		4-chloro-2-methylphenol (4C2MP)	1570-64-5
		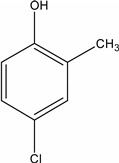	
Mecoprop (2-(4-chloro-2-methylphenoxy) propanoic acid)	7085-19-0	4-chloro-2-methylphenol (4C2MP)	1570-64-5
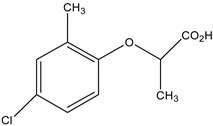		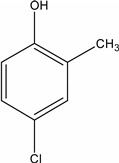	
Mecoprop-p ((2-R)-2-(4-chloro-2- methylphenoxy)propanoic acid)	16484-77-8	4-chloro-2-methylphenol (4C2MP)	1570-64-5
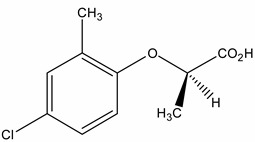		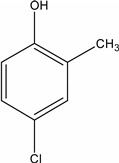	
2,4-D ((2,4-dichlorophenoxy)acetic acid)	94-75-7	Phenoxyacetic acid (PAC)	122-59-8
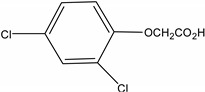		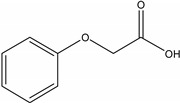	
		2,4-Dichlorophenol (DCP)	120-83-2
		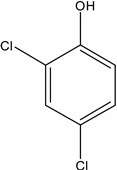	
Bromoxynil (3,5-dibromo-4-hydroxy-benzonitrile)	1689-84-5	3,5-Dibromo-4-hydroxybenzoic acid (BrAc)	3337-62-0
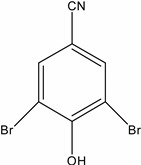		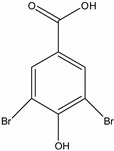	
Triclopyr ([3,5,6-trichloropyridinyl)oxy] acetic acid)	55335-06-3	3,5,6-Trichloro-2-pyridinol (T2P)	6515-38-4
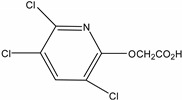		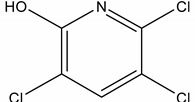	
TBA (2,3,6-trichlorobenzoic acid)	50-31-7	2,4,5-Trichlorophenol (TCP)	95-95-4
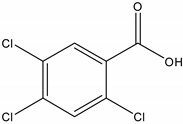		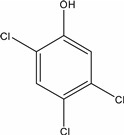	
Dichlobenil (2,6-dichlorobenzonitrile) *	1194-65-6	2,6-Dichlorobenzamide (BAM)	2008-58-4
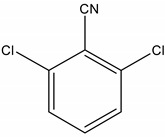		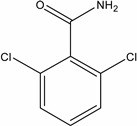	
		3,5-dichlorobenzoic acid (DBA)	50-30-5
		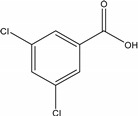	
Bentazone (3-(1-methylethyl)-1*H*-2,1,3-benzothiadiazin-4(3*H*)-one 2,2-dioxide ^†^	25057-89-0		
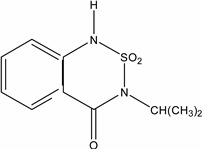			
Dicamba (3,6-dichloro-2-methoxybenzoic acid) ^†^	1918-00-9		
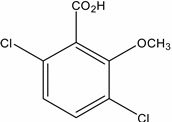			
Fluroxypyr [(4-amino-3,5-dichloro-6-fluoro-2-pyridinyl)oxy]acetic acid) ^†^	69377-81-7		
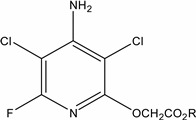			

* Compound not analysed using method presented here; ^†^ Compound has no TP analysed by the method presented here.

This paper describes the development and validation of a method to determine ten phenoxyacetic acid herbicides and six of their most common TPs in groundwater along with two benzonitrile TPs. This method is advantageous over existing published methods for phenoxyacetic acid herbicide residues in groundwater because of the wider number of compounds included, the simplicity of the sample preparation procedure and sensitivity. In addition, both negatively and positively charged analytes are detected by a single UHPLC-MS/MS run, which shortens analysis time and uses less solvent. This paper applies the method described to determine these 18 analytes in Irish groundwater for the first time.

## 2. Results and Discussion

### 2.1. Method Development

#### 2.1.1. UHPLC-MS/MS Conditions

The analysis of phenoxyacetic acids and their TPs is complicated because of the structural similarity of these molecules. In addition, they form negatively charged ions in electrospray ionisation (ESI) while the benzonitrile TP BAM forms positively charged ions. Therefore, chromatography and mass spectrometry conditions had to be carefully optimised. The best chromatographic separation and MS signals were achieved using water and acetonitrile both containing 0.01% formic acid. The addition of formic acid was necessary to improve peak shape and enhance chromatographic retention. The sensitivity for the analyte 2,4,5-TCP was found to decrease significantly with formic acid addition. In contrast, modifying the formic acid concentration from 0.001% to 0.01% increased the signal-to-noise ratio (S:N) for PAC from 69 to 173, respectively. Ammonium formate was evaluated as a mobile phase additive but depressed each analyte’s signal in both ionisation modes, thus formic acid was chosen at 0.01% v/v. DMSO, methanol, acetonitrile, methanol/water (50:50,v/v) and acetonitrile/water (50:50, v/v) were evaluated as injection solvents. Acetonitrile/water (50:50, v/v) was selected as the injection solvent because it gave the best response for all compounds, with increased S:N for the less sensitive compounds PAC and DBA. It is important that modern analytical methods both accurately measure and qualitatively identify target analytes at low concentrations in water samples. As PAC was one of the least responsive analytes, the method was optimised for PAC thus 0.01% formic acid was chosen as the mobile additive. Two different transitions were identified for the majority of analytes by selecting alternative precursor or product ions during the low energy collision induced dissociation optimisation experiments. However, only one transition was identified for BrAc and mecoprop(s) (Table 2). In addition, 4C2MP and T2P did not produce any product ions but two and three precursor ions were identified, respectively. The selectivity of transitions was evaluated through the injection of individual standards and monitoring for cross-talk and isobaric interference in UHPLC-MS/MS traces. Mecoprop and mecoprop-p both had the same transition (213.1 → 140.9) but could not be chromatographically resolved. Mecoprop-p has probably been more prevalent in the Irish environment since 2001 following a decline in mecoprop marketing and usage.

Prior to any sample run the column was allowed to heat up to temperature and the difference in mobile phase pressure was allowed to stabilise. A system suitability check was first tested to confirm all analytes of interest were eluting at the correct retention time. Each analyte was checked to make sure their confirmatory ion was present in the suitability check prior to beginning a run.

**Table 2:** UHPLC-MS/MS conditions for the analysis of herbicides in water.

Compound	Empirical Formula	MW ^1^ (g/mole)	Transition (*m/z*)	Cone (V)	CE ^2^ (V)	Dwell Time(s)	ESI Polarity	SRM Window ^3^
Phenoxyacetic acid herbicides								
PAC	C_8_H_8_O_3_	152.2	151.9 → 94.1	23	14	0.07	Neg.	2
			151.9 → 108.0	23	9	0.07		
Dicamba	C_8_H_6_Cl_2_O_3_	221.0	219.0 → 175.0	15	8	0.07	Neg.	2
			221.1 → 176.8	15	7	0.07		
TBA	C_7_H_3_Cl_3_O_2_	225.5	223.0 → 178.9	14	7	0.07	Neg.	2
			224.9 → 180.9	14	7	0.07		
Bentazone	C_10_H_12_N_2_O_3_S	240.3	239.2 → 132.0	26	27	0.07	Neg.	3
			239.2 → 175.0	26	20	0.07		
			239.2 → 196.9	26	21	0.07		
Fluroxypyr	C_7_H_5_Cl_2_FN_2_O_3_	255.0	253.0 → 194.6	22	13	0.07	Neg.	3
			253.0 → 232.9	22	4	0.07		
BrAc	C_7_H_4_Br_2_O_3_	295.9	294.9 → 250.8	35	18	0.005	Neg.	3
DCP	C_6_H_4_Cl_2_O	162.9	160.8 → 125.0	36	16	0.02	Neg.	4
			162.9 → 127.0	36	19	0.02		
T2P	C_5_H_2_Cl_3_NO	198.4	196.0 → 196.0	28	1	0.02	Neg.	4
			197.9 → 197.9	28	1	0.02		
			199.9 → 199.9	28	1	0.02		
MCPA	C_9_H_9_ClO_3_	200.6	199.1 → 141.0	26	15	0.015	Neg.	4
			201.1 → 143.0	26	15	0.015		
MCPA D6 (methyl-D3, phenoxy-D3)	C_9_H_9_ClO_3_D_6_	206.6	204.9 → 146.9	25	15	0.01	Neg.	4
2,4-D	C_8_H_6_Cl_2_O_3_	221.0	219.0 → 160.9	24	16	0.015	Neg.	4
			220.9 → 162.9	24	15	0.015		
Phenoxyacetic acid herbicides																
Triclopyr	C_7_H_4_Cl_3_NO_3_	256.5	254.0 → 195.9	19	14	0.015	Neg.	4
			254.0 → 218.1	19	6	0.015		
Bromoxynil	C_7_H_3_Br_2_NO	276.9	276.0 → 78.9	38	26	0.015	Neg.	4
			275.9 → 80.9	38	26	0.015		
4C2MP	C_7_H_7_ClO	142.6	141.0 → 141.0	36	5	0.1	Neg.	4
			143.0 → 143.0	36	5	0.1		
TCP	C_6_H_3_Cl_3_O	197.5	196.9 → 159.0	25	18	0.08	Neg.	5
			196.9 → 160.9	25	18	0.08		
Mecoprop	C_10_H_11_ClO_3_	214.7	213.1 → 140.9	25	16	0.1	Neg.	5
Mecoprop-p	C_10_H_11_ClO_3_	214.7	213.1 → 140.9	25	16	0.1	Neg.	5
Benzonitrile herbicides																
BAM	C_7_H_5_Cl_2_NO	190.0	190.0 → 109.0	34	34	0.15	Pos.	1
			190.0 → 144.9	34	27	0.15		
			190.0 → 172.9	34	18	0.15		
DBA	C_7_H_4_Cl_2_O_2_	190.0	188.8 → 144.8	25	11	0.015	Neg.	5
			190.8 → 147.0	25	11	0.015		

^1^ MW: Molecular weight; ^2^ CE: Collision energy; ^3^ SRM (selected reaction monitoring) 1 (2.5–3.14 min); SRM 2 (2.8–4.0 min); SRM 3 (3.8–4.75 min); SRM 4 (4.7–5.73 min); SRM 5 (5.3–8.0 min).

#### 2.1.2. Sample Preparation Procedure

The objective was to develop a robust sample preparation procedure for the isolation of herbicide residues from water samples. In recent years, polymeric sorbent materials have been marketed as an alternative to traditional alkyl bonded silicas. Polymeric sorbents are advantageous because they are do not lose adsorption capacity when dried out or working with aqueous samples, do not suffer from residual silanol effects that occur with silica and require a lower bed mass. In this work, a range of different SPE cartridges were initially evaluated, including Strata-X 33 µm polymeric sorbent (100 mg/3 mL and 200 mg/6 mL), Strata-XL 100 µm polymeric reversed phase 100 mg/3 mL, Strata SDB-L Styrene-divinylbenzene polymer 500 mg/3 mL (all available from Phenomenex, Macclesfield, UK), Bond Elut ENV 200 mg/6 mL (Agilent, Cork, Ireland), and Oasis^®^ HLB 200 mg/6 mL (Waters, Dublin, Ireland).

**Figure 1:**
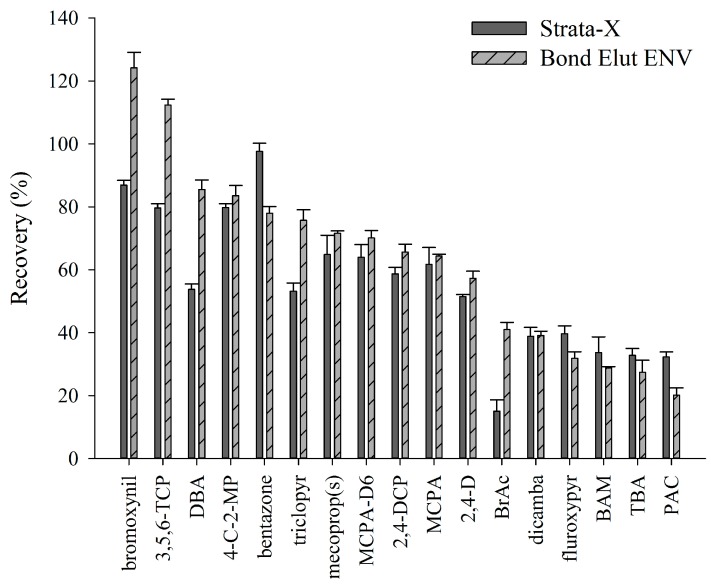
Recoveries during method development for each compound using samples fortified at the European Union Drinking Water Standard of 0.1 µg·L^−1^ from Strata-X and Bond Elut ENV (200 mg in 6 mL) SPE cartridges. Error bars represent standard error of the sample mean.

Bond Elut ENV 200 mg/6 mL and Strata-X 100 mg/6 mL cartridges gave the best overall recovery results and most consistent flow. These cartridges were selected for further optimisation using 500 mL water samples fortified with 200 µL of a 500 ng·mL^−1^ standard to give 0.2 μg·L^−1^. Acidifying the sample prior to loading onto the SPE cartridge achieved better peak shape and absorption of compounds of interest onto the sorbent bed. All water samples, including validation samples, were spiked with 2 mL of formic acid to achieve a concentration of 0.1% prior to SPE. Tabani *et al.* [23] also found that sample pH influenced SPE sorption because at pH values less than 4.8, chlorophenoxy acid herbicides can interact through hydrogen bonding between their carboxylate groups. Cartridges were initially eluted with 6 mL of acetonitrile. Recovery ranged from 15% to 86% for Strata-X and 20%–124% for Bond Elut ENV (Figure 1). Recovery should be in the range 70%–120% for groundwater matrices [24]. As a result of these poor recoveries, a range of different elution solvents were evaluated including acetone, chloroform, acetonitrile/water (50:50, v/v), methanol, methanol/water (50:50, v/v), and MTBE/methanol (90:10, v/v). Particular, attention was given to the improvement of recovery for the analytes showing poor recovery and low sensitivity on the UHPLC-MS/MS, namely, PAC, TBA, BAM, fluroxypyr, dicamba, and BrAc. An elution solvent comprising 6 mL of acetone was found to give the best overall recovery, but there was a compromise with a decrease in recovery for 3,5,6-TCP. Different volumes of acetone elution solvent (6, 7, and 8 mL) were evaluated and optimal recovery was achieved with 7 mL (average of 86% for all 18 compounds). Evaporation temperatures were subsequently evaluated (30, 35, 40, and 45 °C) with 40 °C found to be optimum.

### 2.2. Method Validation

#### 2.2.1. Recovery, Precision, Limit of Detection and Calibration

The recovery and precision of the method was evaluated during a repeatability experiment over three days. The recovery ranged between 71% and 118% for all 18 compounds (Table 3).

**Table 3:** Validation study results for accuracy and precision tested at three concentrations: 0.02, 0.04 and 0.06 µg·L^−1^, the calculated limit of detection (LOD) and limit of quantification (LOQ). Accuracy and precision was determined from six replicates carried out for each validation level.

Analyte	Validation Level (μg·L^−1^)						LOQ (µg·L^−1^)	LOD (µg·L^−1^)
	Accuracy (%)			Precision (%RSD)				
	0.02	0.04	0.06	0.02	0.04	0.06		
BAM	95	105	88	18	12	22	0.0009	0.0006
PAC	110	105	109	18	2	13	0.0063	0.0015
Dicamba	106	116	108	13	11	10	0.0004	0.0003
TBA	97	111	94	18	9	16	0.0517	0.0047
Bentazone	112	101	97	23	8	10	0.001	0.00009
Fluroxypyr	92	118	99	30	5	15	0.0007	0.0002
BrAc	101	99	79	16	13	15	0.0023	0.0018
DCP	109	103	99	16	8	5	0.0014	0.0007
T2P	113	109	94	8	9	10	0.0306	0.0025
MCPA	109	90	71	32	20	18	0.0003	0.0001
2,4-D	112	107	98	14	8	5	0.0005	0.0003
Triclopyr	113	108	102	15	8	8	0.0023	0.0004
Bromoxynil	112	106	94	16	15	17	0.0015	0.0002
4C2MP	100	109	103	46	5	25	0.0002	0.0001
DBA	103	96	88	45	42	41	0.0625	0.0036
TCP	105	108	97	12	7	9	0.0049	0.0012
Mecoprop(s)	115	96	102	19	16	11	0.0002	0.00008

The precision (measured as the percentage relative standard deviation (%RSD)) was less than 32% for all compounds except 4C2MP at the 0.02 µg·L^−1^ level: 46%RSD. The lower precision for 4C2MP is attributed to laboratory work carried out on day two of the study. Removing results from day 2 improves the overall precision to 25.5%. DBA also had a lower %RSD across all three concentration levels: from 45% RSD at 0.02 µg·L^−1^ to 41% RSD at 0.06 µg·L^−1^ levels. These values are low because the calculated LOQ for DBA is 0.0625 µg·L^−1^ and the fortified concentrations were lower at 0.02, 0.04, and 0.06 µg·L^−1^. The linearity of the method was evaluated over each curve with the range 0.008–0.4 µg·L^−1^ and 0.4–4.0 µg·L^−1^ during each calibration run. The RSD between replicates and any calibration residuals were assessed to reduce potential bias. The coefficient of determination (measured as r^2^ values) were accepted when they were greater than 0.995. The LOD and LOQ calculated from six fortified samples at the concentration 0.02 µg·L^−1^ can be found for each analyte of interest in Table 3.

#### 2.2.2. Stability of Analytes in Water Samples

**Figure 2:**
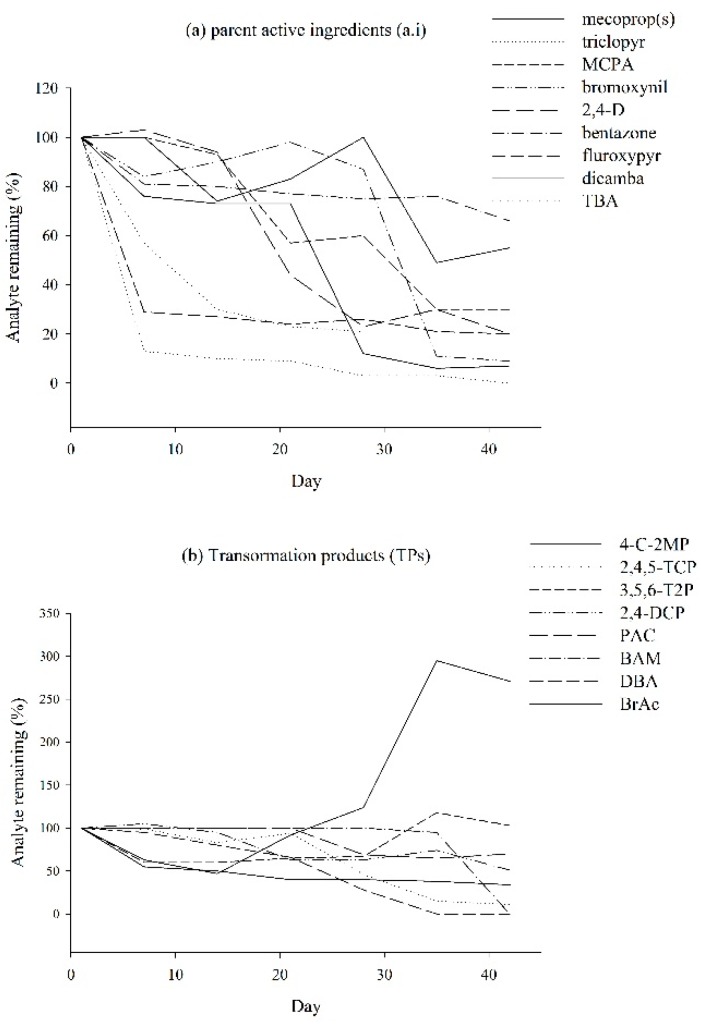
The stability of raw groundwater samples every seven days over a 42 d period using blank groundwater fortified to achieve 0.1 µg·L^−1^. Results are expressed as a percentage of the analyte remaining after day one. (**a**) parent phenoxyacetic acid herbicides and (**b**) transformation products (TPs).

The stability of raw groundwater samples is shown in Figure 2. The most unstable compounds are TBA followed by fluroxypyr, triclopyr, BrAc, and 4C2MP. The concentration of 4C2MP begins to increase after day 14. MCPA begins to drop in concentration on day 14 and mecoprop(s) fluctuates in concentration between day 14 and day 28 before decreasing on day 35, which coincides with a sharp increase in 4C2MP. BAM, T2P, DCP, and PAC remain the most stable compared to the other compounds. This study shows that following collection and chilled storage at 4 °C, samples should be analysed within seven days or less, otherwise compounds such as TBA, fluroxypyr, and triclopyr will drastically reduce in concentration, while 4C2MP will increase in concentration after 14 days.

#### 2.2.3. Comparison with Published Methods

The current method is compared with several other published methods for similar compounds in Table 4. Rodil *et al.* [21] developed a method to quantify 53 compounds; six were phenoxyacetic acid herbicides. Similar compounds were mecoprop and 2,4-D with LOD of 0.0025 and 0.0006 µg·L^−1^ respectively, during a 41 min run time. This method does not quantify for as great a number of analytes across several chemical classes, but is able to detect the most number of phenoxyacetic acid herbicides in comparison to other similar methods [3,17,21,23,25,26]. The LOD for each compound analysed using this method was between 0.00008 and 0.0047 µg·L^−1^ (Table 3). The higher LOD for some of the phenoxyacetic acids, e.g. TBA and DBA, could be attributed to interactions along the stationary phase during laminar flow of the HPLC process [23].

**Figure 3:**
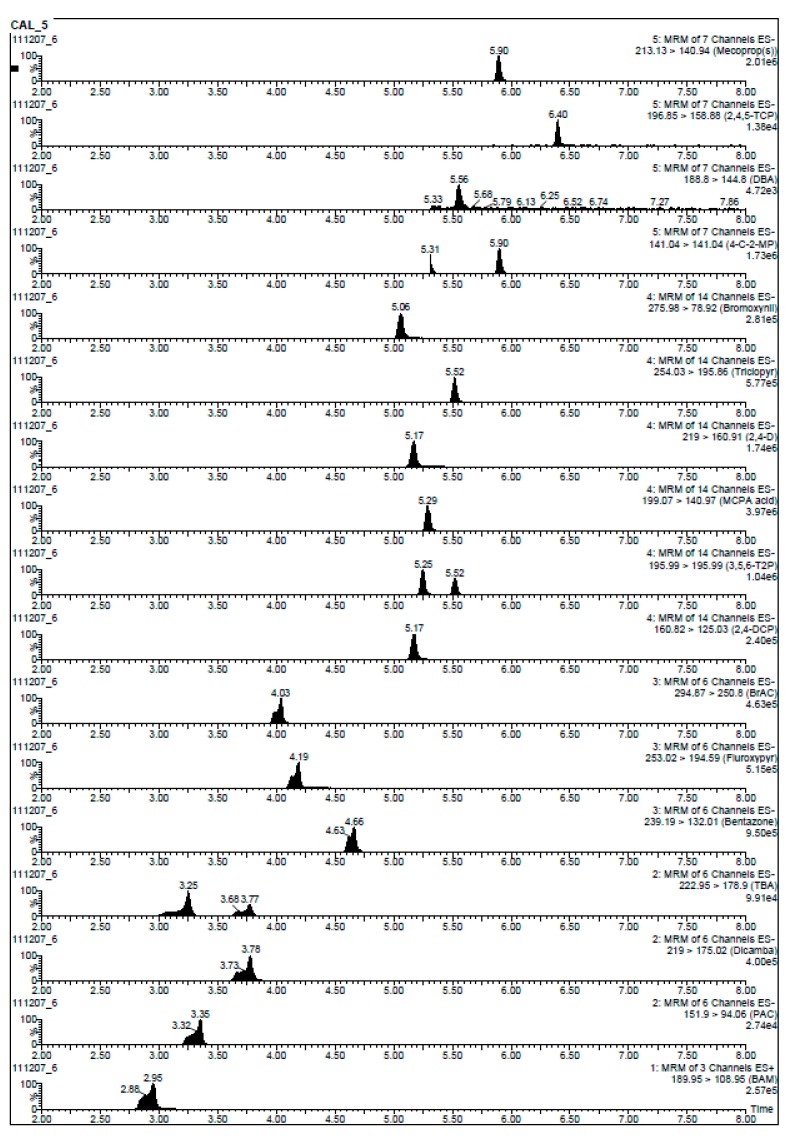
UHPLC-MS/MS trace for all 18 compounds at 0.4 µg·L^−1^ after SPE clean up and re-suspension in acetonitrile/ultra pure water (50:50, v/v).

The peak shape for analytes eluting in the first 5 mins of the chromatogram was poorer compared to later eluting peaks (Figure 3). This is largely due to the injection volume of 20 µL and injection solvent used. In newer mass spectrometers, lower injection volumes can be used, which will negate this band broadening effect and give sharper peaks.

**Table 4:** Comparison on analytical performance of method presented here with other methods which also analyse for phenoxyacetic acid herbicides.

Author(s)	Separation	Detection	Extraction	Range of Analytes	Number of Phenoxyacetic Acid Herbicides	Separation Time (min)	Sample Size (mL)	Recovery Range (%)	Detection Limit (µg·L^−1^)
LOD from this method	UHPLC	MS/MS	SPE	18	16	11.9	500	88–118	0.00008–0.0047 (refer to Table 3)
Rodil *et al.* [21]	LC	MS/MS	SPE	53	6	41	200	22–146	0.0006 (2,4-D)0.0025 (mecoprop)
Marin *et al.* [25]	UHPLC	MS/MS	SPE	37	3	10	100	70–120	0.025
Marchese *et al.* [17]	LC	MS/MS	Graphite cartridge	8	4	c.15	500	85–103	0.0001
Solymosné Majzik *et al.* [26]	LC	MS/MS	SPE	6	4	15	500	76–108	0.0011 (dicamba)0.0017 (2,4-D)0.0029 (MCPA)0.0015 (mecoprop)
Sklivagou *et al.* [3]	LC	MS/MS	SPE	6	3	15	500	61–120	0.03 (2,4-D)0.003 (MCPA)0.003 (bentazone)
Tabani *et al.* [23]	CE ^†^	UV ^‡^	SPE-EME *	3	3	20	100	75–77	1.0 (mecoprop)1.5 (MCPA)

^†^ Capillary electrophoresis; ^‡^ Ultra violet; * Electro membrane extraction.

Future research could include the development of methods which can distinguish between mecoprop and mecoprop-p even though they have the same transitions required for mass spectrometry identification. Chiral chromatography may be a solution. Future methods could also determine how long the column needs to equilibrate to improve peak shape and which injection volume is best suited.

### 2.3. Application to Environmental Groundwater Samples

**Figure 4:**
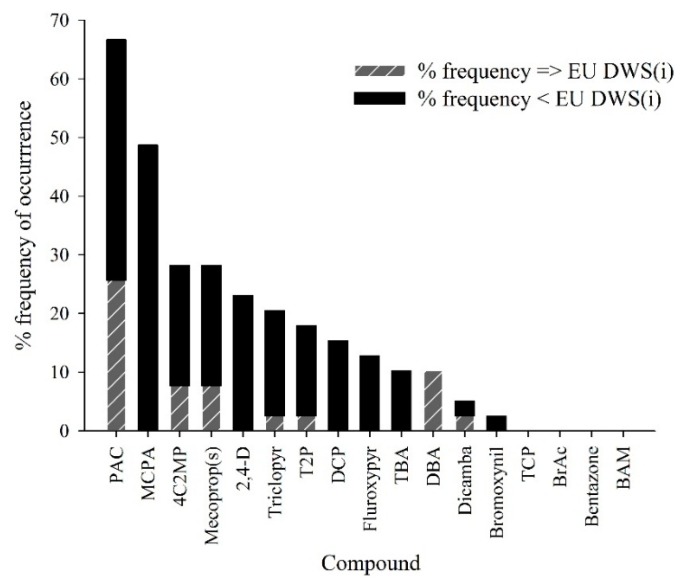
Percentage frequency of occurrence of compounds detected at concentrations either in breach of the European Union drinking water standard for individual compounds (EU DWS(i)) of 0.1 µg·L^−1^ or present in a detectable concentrations in groundwater from 42 samples collected at seven locations across Ireland in March 2012.

**Figure 5:**
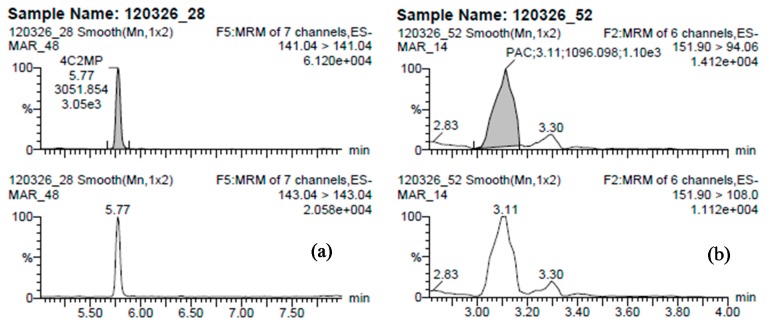
Selected UHPLC-MS/MS chromatograms from groundwater samples collected from an intensive agricultural area in Ireland. Concentrations of (**a**) 4C2MP [0.029 µg·L^−1^] and (**b**) PAC [1.6 µg·L^−1^].

The method developed was applied to 42 groundwater samples collected in March 2012 from seven locations across Ireland. Figure 4 shows the frequency of occurrence of the compounds collected in groundwater. Thirteen compounds were detected in groundwater collected. Eight of the compounds detected were parent active ingredients and five were TPs. Compounds detected in concentrations exceeding the EU drinking water limit for individual pesticides (0.1 µg·L^−1^) were (in decreasing frequency of occurrence): PAC, DBA, 4C2MP, mecoprop(s), dicamba, triclopyr, and T2P. Summary statistics of all analytical results from the 42 groundwater samples collected in March 2012 and analysed using the current method is presented in Table 5 and the physico-chemical groundwater characteristics of samples collected across the seven sites are presented in Table 6. Samples which exceeded the highest calibration standard were reanalysed and if still higher than 0.4 µg/L were reanalysed using a high calibration curve ranging from 0.4 to 4 µg/L. The most frequently detected compounds were PAC and MCPA present in 67% and 49% of the 42 samples collected in March 2012, respectively. Figure 5 shows the chromatogram of a positive sample for PAC and 4C2MP. Fava *et al.* [27] and Hotlze *et al.* [9] indicate that DBA is a TP of dichlobenil, with BAM and DBA are formed in the environment from the hydrolysis of the nitrile group of the parent herbicide dichlobenil and then subsequent hydroxylation at the three-position of the phenyl ring [28]. The source of BAM is most likely from dichlobenil, which was previously applied to surface water courses in Ireland by Waterways Ireland [29]. The application of dichlobenil has since ceased following its removal from the Irish market in 2007 [30].

**Table 5:** Minimum, maximum, median and average pesticide concentrations (µg·L^−1^) of groundwater samples collected in March 2012. (LOD and LOQ for each pesticide are presented in Table 3).

Compound	Minimum	Maximum	Median	Average
2,4-D	0.002	0.007	0	0.001
4C2MP	0.005	1.364	0.005	0.076
BAM	<LOD	<LOD	0	-
Bentazone	<LOD	<LOD	0	-
BrAc	<LOD	<LOD	0	-
Bromoxynil	0.008	0.08	0	0.003
DBA	3.019	14.218 *	0	1.172
DCP	0.001	0.004	0	0.001
Dicamba	0.003	0.126	0	0.005
Fluroxypyr	0.003	0.004	0	0.001
MCPA	0.005	0.01	0.006	0.004
Mecoprop(s)	0.006	1.461	0.006	0.079
PAC	0.015	4.148 *	0.216	0.456
T2P	0.037	0.146	0	0.012
TBA	0.005	0.026	0	0.002
TCP	<LOD	<LOD	0	-
Triclopyr	0.023	0.15	0.001	0.013

* extrapolated concentration.

The presence of PAC may be from the degradation of MCPA or 2,4-D [31] or PAC may be an impurity within MCPA or 2,4-D formulation products [32]. Although the degradation pathway has not been proven, it is highly likely that when MCPA or 2,4-D degrade, they will degrade to PAC (Table 1). Vroumsia *et al.* [33] states that PAC is the non-chlorinated version of 2,4-D so should the chlorines present in 2,4-D degrade in the environment, the end product will be PAC.

**Table 6:** Description of groundwater samples collected from seven sites across Ireland in March 2012. Values are averaged across all samples collected at that site on each day.

Site Name	Number of Samples Analysed	Number of Replicates across Site Collected and Analysed	Sample Date	pH	Redox (mV)	Conductivity (µS/cm @ 25 °C)	Turbidity (NTU)	Temperature (°C)
KWDg	2	2	13th March	7.2	189	595	0	8.4
KWDa	2	2	21st March	7.9	113	470	0	10.2
I/KWDa	7	2	13th March	7.9	180	406	128	9.6
FvWDa	3	2	14th March	6.1	164	127	167	9.5
FvPDa	9	2	14th March	7.3	-40	263	194	9.8
FmWDa_1_	10	2	21st March	7.1	117	220	28	9.7
FmWDa_2_	9	2	20th March	6.5	120	196	53	10.6

The chlorophenol TP 4C2MP has been proven by Zertal *et al.* [34,35] as a higher toxicity TP than the parent a.i. it was derived from: MCPA. Vione *et al.* [36] state that 4C2MP can form in surface waters following direct photolysis of MCPA. Mecoprop (both mecoprop and mecoprop-p) can degrade to 4C2MP [37] in laboratory cultures [38], soils [39], and groundwater [40,41]. The present study found during a stability test that the concentration of 4C2MP began to increase on day 14 of the raw groundwater stability experiment (Figure 2). MCPA concentration decreased on day 14 while mecoprop(s) concentration fluctuated in concentration between day 14 and 28 before sharply decreasing on day 35. 2,4-D and dicamba also reduced in concentration while the concentration of 4C2MP increased. This coincides with a sharp increase in 4C2MP (Figure 2) indicating that prolonged sample storage will cause an increase in TP occurrence due to degradation within an amber glass bottle as opposed to degradation in groundwater.

## 3. Experimental Section

### 3.1. Reagents and Materials

Formic acid (FA) and hydrochloric acid (HCl) were purchased from Sigma Aldrich (Arklow, Ireland). Ultra-pure water (18.2 MOhm) was produced in-house using a Millipore water purification system (Cork, Ireland). Acetonitrile, acetone, chloroform, methyl tert-butyl ether (MTBE) and methanol used were all of HPLC grade and purchased from Lennox Laboratory Supplies Ltd. (Dublin, Ireland). Bond Elut ENV (200 mg/6 mL) and Strata-X (200 mg/6 mL) cartridges were from Agilent (Dublin, Ireland) and Phenomenex (Cheshire, UK), respectively. Nitrogen (99% purity) for use in sample concentration was purchased from BOC gases (Dublin, Ireland). Mobile phases were filtered through GH Polypro hydrophilic polypropylene membrane filters 0.2 µm, 47 mm from PALL-company (Ann Arbor, MI, USA). Solvent reservoir caps and Teflon tubing with an I.D of 3 mm were purchased from Waters (Dublin, Ireland). Amber Pyrex^®^ glass sampling bottles (0.5 L) were obtained from Lennox and polyethylene adaptor caps with Luer tips were purchased from Phenomenex. A Techne DB-3 Dri-block^®^ sample concentrator fitted with aluminium blocks, both from Lennox and a vortex mixer from VWR (Dublin, Ireland) were used during sample preparation.

### 3.2. Standard Solutions and Calibration

Analytical standards were purchased from Ehrenstorfer GmbH (Augsburg, Germany) including deuterated MCPA-D_6_ for use as an internal standard. 3,5-dibromo-4-hydroxybenzoic acid (BrAc) was purchased from Wako Chemicals GmbH (Nuess, Germany). Primary stock standard solutions were prepared in HPLC grade methanol at concentrations of 100 µg mL^−1^. Mixed working standards were prepared in HPLC grade methanol at concentrations of 20, 50, 100, 500, and 1000 ng·mL^−1^. MCPA-D_6_ was prepared at concentrations of 500 ng·mL^−1^ in acetonitrile. Deuterated MCPA-D_6_ was used as an internal standard for mecoprop, mecoprop-p, MCPA, 2,4-D, dicamba, triclopyr, fluroxypyr, bromoxynil, bentazone, and PAC. All other compounds used external standards. Primary and working standards were stable for at least six months when stored at 4 °C. Two sets of extracted matrix matched calibrations were prepared by fortifying 500 mL of ultra-pure water with 200 µL of working standard mixes prior to extraction to achieve a low calibration curve ranging from 0.008 to 0.4 µg·L^−1^ and a high calibration curve ranging from 0.4 to 4 µg·L^−1^.

### 3.3. Quality Control

Recovery controls were prepared by spiking four blank samples post-extraction, two with working standard 50 ng·mL^−1^ (50 µL) and two with working standard 1000 ng·mL^−1^ (50 µL) to monitor for loss of analytes during extraction. A negative and positive quality control spike was processed with every batch of water samples extracted using a solid phase extraction (SPE) manifold purchased from Phenomenex. The positive quality control samples were spiked to achieve the concentration 0.1 µg·L^−1^. Negative quality control spikes were prepared as reagent blanks alongside all other samples and standards for analysis. Analytical results were not corrected for recovery because they were analysed using a matrix extracted calibration curve.

### 3.4. Sample Preparation

Water samples (500 mL) in amber glass Pyrex^®^ bottles were acidified by addition of 2 mM HCl (2 mL). 200 µL of the working internal standard solution was then added. The samples were gently agitated and the bottle fitted with a reservoir cap and 40 cm of Teflon^®^ tubing which was connected to Bond Elut ENV SPE cartridges fitted with polyethylene adaptors. Cartridges were preconditioned with 10 mL of HPLC grade methanol and 10 mL of ultra-pure water. Samples were loaded at 5 mL min^−1^ through the cartridges. Once all samples had loaded through the cartridges, adaptor caps and Teflon^®^ tubing were removed. Cartridges were then sequentially eluted by adding 2 × 3.5 mL volumes of HPLC grade acetone into a 14 mL glass test-tube. The acetone was evaporated under nitrogen at 40 °C to dryness. Concentrated extracts were resuspended in 500 µL of acetonitrile/water (50:50, v/v), vortexed for 30 s and filtered through 0.2 µm, 40 mm GH Polypro membrane filters into 2 mL autosampler vials fitted with 200 µL glass inserts. Samples prepared to optimise the SPE clean-up step consisted of 500 mL of ultra-pure water fortified to achieve a concentration of 0.2 µg·L^−1^. Fortified samples were prepared daily for clean-up optimisation work.

### 3.5. UHPLC-MS/MS Conditions

Separations were performed using a Waters (Milford, MA, USA) Acquity UHPLC^®^ system comprising of a stainless steel BEH analytical column (2.1 mm × 100 mm, particle size 1.7 µm) and a 2.1 mm × 10 mm guard column containing the same packing material, both maintained at 60 °C. Mobile phase was pumped at a flow rate of 0.6 mL·min^−1^. A binary gradient separation was used to separate analytes comprised of mobile phase A: 0.01% formic acid in ultra-pure water and mobile phase B: 0.01% formic acid in acetonitrile. The gradient programme was as follows: (1) 0.01 → 1.0 min, 99.9% A; (2) 2 min, 85% A; (3) 7 min, 40% A; (4) 8.5 min, 0.1% A; (5) 9.0 min, 0.1% A and (6) 9.1 → 11.9 min, 99.9% of A. The total run time was 11.9 min and the injection volume was 20 µL. Weak and strong autosampler needle washes consisted of water/acetonitrile (50:50, v/v) and acetonitrile, respectively.

Analytes were detected using a Waters Quattro Premier XE triple quadrupole instrument operating in electrospray ionisation (ESI) mode (Waters). Nitrogen (99.9%) was used for desolvation (1000 L·h^−1^) and cone gas (50 L·h^−1^). Argon (99%) was used as a collision gas (0.013 L·h^−1^). The source and desolvation gas temperatures were set at 130 °C and 350 °C, respectively. The electrospray voltage was set at 3000 eV and 500 eV for positive and negative modes, respectively. The UHPLC-MS/MS system was controlled using MassLynx™ software and data processed using TargetLynx™ software both supplied by Waters.

MS conditions were optimised by tuning analyte-specific parameters such as cone voltage and collision energy. Optimisation was carried out by direct infusion of individual standard solutions at concentrations of 1000 ng·mL^−1^ with mobile phase. The two most abundant product ions produced from the precursor ion were monitored and recorded. The monitored ions and optimised MS conditions for each compound are reported in Table 2.

### 3.6. Validation Procedure

The method was validated in accordance with SANCO/10232/2006 on the guidance of pesticide residue analytical methods [24]. The following validation parameters were investigated: precision, recovery, limit of detection, limit of quantification and linearity. Throughout validation retention times, signal-to-noise ratios (S:N) and ion ratios were monitored to assess the method’s robustness. Validation was carried out at a low level to reflect anticipated concentrations in environmental groundwaters and below permitted limits in the EU drinking water directive [1]. Validation spiking solutions were prepared at 50, 100 and 150 ng·mL^−1^ in HPLC grade methanol which were then spiked into ultra-pure water to give concentrations of 0.02, 0.04 and 0.06 µg·L^−1^ in water, respectively. Precision and recovery was tested by fortifying 500 mL of ultra-pure water at these three different concentrations: (*n* = 6 each concentration) and repeating on three separate days. From these samples the relative standard deviation (RSD) indicated precision.

The stability of raw groundwater samples over time was assessed by spiking 500 mL of groundwater (known to contain undetectable quantities of the compounds of interest) to achieve a concentration of 0.1 µg·L^−1^. Samples were stored in the dark at 4 °C and analysed every seven days over a 42 day period. Compound stability was plotted over time as a percentage of the analytes concentration when freshly prepared at inception of the stability study.

## 4. Conclusions

Using UHPLC-MS/MS with rapid polarity switching, a quantitative multi residue method to determine ten active ingredients present in pesticide product formulations, six phenoxyacetic acid herbicide transformation products, and two benzonitrile transformation products has been developed and validated in accordance with SANCO/10232/2006 criteria. Recoveries ranged between 71% and 118% and limits of detection for the analytes were between 0.000008 and 0.0047 µg·L^−1^.

This is the first method to attempt such a large range of phenoxyacetic acid herbicides and their transformation products in groundwater and to reach detection limits below EU permitted concentrations allowed in groundwater [14] and drinking water [1].

Applying this method to 42 groundwater samples collected in March 2012 from several locations across Ireland has revealed that transformation products are just as commonly detected in groundwater as their parent active ingredient counterparts and both are present at some sites in concentrations in breach of European Directives. It is hoped this study will increase awareness of herbicides in groundwater and their transformation products.
